# Supplementary material for: Association of parental characteristics and emotion regulation in children and adolescents with and without psychopathology: A case-control study
Source: PLoS One. 2022 Jul 27;17(7):e0271486. doi: 10.1371/journal.pone.0271486 (PMC9328518; doi:10.1371/journal.pone.0271486)
Supplement: S1 File — (PDF) [file pone.0271486.s001.pdf]

Supplement

As gender and age of the children seem to influence ER and parenting behavior, we calculated the different analyzes with gender and age as supplemental variables in an explanatory fashion and reported this in S1-File. Regarding gender differences and age no specific hypotheses were made because research concerning age and gender is inconsistent.

Results

Hypothesis 1

As matching led to a significantly smaller sample size, we performed the analyses with the original sample (n = 229) in an exploratory fashion to check for the influence of age and gender. As groups were no longer dependent but differed in age, we used a MANCOVA with gender as a supplemental factor and with age as a covariate. Using Pillai's trace there was a significant effect for group (V =.09, F(2,223) =10.39, p <.001.) but not for the covariate age (V = .01, F(2,223) = .74, p = .478). No effect for gender (V = .004, F(2,223) = .45) p = .639) and no interaction effect gender*group (V = .003, F(2,223) = .37 , p = .692) was found. Post hoc ANOVA revealed a significant effect for reappraisal (F(1,224))= 6.85, p = .009). There was also found a significant effect for suppression (F(1,224) = 11.33, p =.001). Including the additional factors didn't change the results.

Hypothesis 2

As matching led to a significantly smaller sample size and to shed light on the influence of age and gender of the children on the parenting behavior, we performed the analyses with the original sample in an exploratory fashion again. As groups were no longer dependent but differed in age, we used a MANCOVA with age as covariate and gender as supplemental factor. The groups did not differ in their reaction also in the original sample (n = 229), introducing gender as a supplemental factor and controlling age as a covariate. Using

Pillai's trace there was no significant effect neither for group (V = 0.006, F(2,223) = 0.63, p =.531) nor for the covariate age (V = 0.001, F(2,223) = 0.01, p = .910) nor for gender (V = .01, F(2,223) = 1.38, p = .254) nor for the interaction effect group*gender (V = .003, F(2,223) = .37, p =.629).

Hypothesis 3

Reappraisal

To test the third hypothesis, we added to the original analysis the factors age and gender as two supplemental predictors. 139 cases could be included like in the original analysis, in two separate hierarchical multiple regressions (reappraisal and suppression). As there were significant deviations from normality in the variable mental health (z_{kurtosis} =12,18; p <0.001; z_{skewness} = 11,75, p <0.001), a bootstrapping procedure using 1,000 samples was used in the hierarchical multiple regressions.

As shown in S1 Table for reappraisal, the first model step including factor age was not significant F(1,137) = .61, p = .437, adjusted R^2 = .003, ΔR^2 = .004. The second step, including gender was also not significant (F2,136) =1.33, p = .268) adjusted R^2 = .005, ΔR^2=.02. The third step parents' reaction to their child's negative emotions was significant, F(4, 134) = 3.75, p = .006, adjusted R^2 = .074, ΔR^2.=.08 The fourth model step including parents' ER was also significant, F(6,132) = 3.38, p = .004 adjusted R^2=.094, ΔR^2=.03. The fifth step including of parents' mental health remained significant, F(7,131) = 22.88, p = .008, adjusted R^2 = .087, ΔR^2 =.001. However, no additional variance could be explained by mental health (see S1 Table). S1 Table shows the standardized and unstandardized regression coefficients with bootstrapped confidence intervals.

S1 Table. Unstandardized *(b)* and Standardized (β) Regression Coefficients for Each Predictor in a Hierarchical Regression Model Predicting Reappraisal in Children

Predictor	*b*	95% CI for *b*		*SE* β	β	R^2	ΔR^2	*p*
		LL	*UL*					
Step 1						.004	.004	.437
Constant	22.39	21.07	23.72	.62				≤.001
z-age	-.54	-1.65	1.04	.70	-.07			.437
Step 2						.02	.02	.155
Constant	19.50	15.27	23.73	2.14				≤.001
gender	1.97	--.75	4.69	1.38	.13			.155
Step 3						.10	.08	.033
Constant	18.54	14.38	22.61	2.83				≤.001
z-CCNES unsupportive	0.69	-.14	5.16	.69	.08			.312
z-CCNES supportive	2.65	0.899	3.63	.69	.27			.001
Step 4						.13	.03	.086
Constant	18.53	14.49	22.63	22.66				≤.001
z-ERQ reappraisal parent	1.46	0.16	.139	2.78	.19			.031
z-ERQ suppression parent	0.34	-.94	1.62	.68	.044			.601
Step 5						.13	.001	.884
Constant	18.54	14.44	22.64	2.73				≤.001
z-GSI mental health parent	-.16	-1.69	1.46	.76	-.012			.884

Note. *N* = 139. CI = Confidence interval (based on 1,000 bootstrapped samples); *LL* = lower limit; *UL* = upper limit; z-age = mean standardized score on age; z-CCNES = mean z-standardized score on the Coping with Children's Negative Emotions Scale; z-ERQ = mean z-standardized score on the Emotion Regulation Questionnaire; z-GSI = mean z-standardized score on the Global Severity Index.

Suppression

A second hierarchical multiple regression was calculated to predict suppression based on age on step 1, gender on step 2, parents' reactions to negative emotions of their children at Step 3, emotion regulation in parents at Step 4, and mental health at step 5. Once again, a bootstrapping procedure based on 1,000 samples was applied. The first factor age was significant (F(1, 137) = 23.61, p≤.001, adjusted R^2 = .14 , ΔR^2 = .19. Including the next four steps no additional variance could be explained (Step2 gender: (F(2,136) = 11.83, p ≤ .001, adjusted R^2 = .14, ΔR^2 =.001, step 3: F(4, 134) = 5.89, p ≤ .001, adjusted R^2 = .12, ΔR^2 = .001. Including emotion regulation strategies of parents did not significantly change the explained variance, F(6, 132) = 4.28, p = .001,. adjusted R^2 =-.12, ΔR^2 =.01. Finally, including mental health of parents did not increase the explained variance, F(7, 131) = 3.61, p

= 001), adjusted R^2 =-.02, ΔR^2 =. 01. S2 Table shows that only the factor age in our model could predict suppression.

S2 Table. Unstandardized *(b)* and Standardized (β) Regression Coefficients for Each Predictor in a Hierarchical Regression Model Predicting Suppression in Children

Predictor	*b*	CI		*SE* β	β	R^2	ΔR^2	*p*
		LL	*UL*					
Step 1						.38	.15	≤.001
Constant	11.54	10.67	12.40	.43				≤.001
z-age	2.22	1.31	3.10	.46	.39			≤.001
Step 2						.39	.001	.660
Constant	10.51	8.18	13.72	1.34				≤.001
gender	.40	-1.39	2.18	.90	.04			.660
Step 3						.39	.001	.891
Constant	10.95	8.12	13.76	.1.42				≤.001
z-CCNES unsupportive	-.16	-.77	1.10	.47	-.03			.729
z-CCNES supportive	.17	-.77	1.03	.47	-.03			.725
Step 4						.40	.012	.405
Constant	10.83	8.03	13.66	1.43				≤.001
z-ERQ reappraisal parents	0.94	.-.82	1.01	.46	.02			.839
z-ERQ suppression parents	0.60	-.30	1.42	.49	.11			.184
Step 5						.40	.001	.715
Constant	10.84	8.00	13.67	1.44				≤.001
z-GSI mental health parent	0.17	-.91	1.25	.49	.02			.804

Note. N = 139. CI = Confidence interval (based on 1,000 bootstrapped samples); *LL* = lower limit; *UL* = upper limit; z-age = mean standardized score on ag; z-CCNES = mean z-standardized score on the Coping with Children's Negative Emotions Scale; z-ERQ = mean z-standardized score on the Emotion Regulation Questionnaire; z-GSI = mean z-standardized score on the Global Severity Index;
